# Type 2 diabetes is associated with an increased prevalence of respiratory symptoms as compared to the general population

**DOI:** 10.1186/s12890-017-0443-1

**Published:** 2017-07-17

**Authors:** F. De Santi, G. Zoppini, F. Locatelli, E. Finocchio, V. Cappa, M. Dauriz, G. Verlato

**Affiliations:** 1 Division of Endocrinology, Diabetes and Metabolism, Department of Medicine, University of Verona, Verona, Italy; 2 Unit of Epidemiology and Medical Statistics, Department of Diagnostic and Public Health, University of Verona, Strada Le Grazie, 8 -, 37134 Verona, Italy

**Keywords:** Dyspnoea, Chronic cough/phlegm, Eczema, Asthma-like symptoms, Type 2 diabetes, Ageing, Obesity, Smoking

## Abstract

**Background:**

To estimate the prevalence of respiratory symptoms in individuals with type 2 diabetes, as compared to the general population.

**Methods:**

Between 2007 and ﻿2010 the screening questionnaire of GEIRD (Gene Environment Interactions in Respiratory Diseases) study was administered to two samples of Verona general population, aged respectively 45-64 years and 65-84 years, and to a convenience sample of individuals with type 2 diabetes, consequently recruited at the local Diabetes Centre. Ninety-four and 165 people with type 2 diabetes, aged respectively 45-64 and 65-84 years, were compared with 676 and 591 subjects in the same age range from the general population. The influence of type 2 diabetes on respiratory symptoms was evaluated by logistic regression models, controlling for sex, age (45-54, 55-64, 65-74, 75-84 years), education level, smoking habits and heavy vehicle traffic exposure and adjusting standard errors of ORs for intra-sample correlation.

**Results:**

Compared to the general population, dyspnoea limiting walking pace on level ground (grade 2 dyspnoea) was more frequently reported by people with type 2 diabetes, irrespective of age (*p* < 0.001), while self-reported chronic cough/phlegm was more common in those aged 45-64 years (*p* = 0.02). These results were confirmed by multivariable analysis: compared to their counterparts from the general population, people with type 2 diabetes aged 45-54 years showed an increased risk of reporting grade 2 dyspnoea (OR = 3.92, 95% CI 3.28-4.68) or chronic cough/phlegm (OR = 1.69, 1.60-1.78). Similar figures held significant at older ages (75-84 years), although partially blunted (dyspnoea: OR = 1.79, 1.68-1.91; chough/phlegm: OR = 1.09, 1.03-1.16). As such, the interaction between age class and type 2 diabetes was significant for both respiratory disorders. The proportion of self-reported dyspnoea among individuals with type 2 diabetes significantly increased across incremental body-mass index (BMI), from 15.4 to 25.4% and further to 41.3% respectively in normoweight, overweight and obese patients (*p* = 0.048).

**Conclusions:**

People with type 2 diabetes more frequently reported grade 2 dyspnoea and chronic cough/phlegm than the general population of the same age, although presenting similar smoking habits. Diabetes appears to anticipate the lung ageing process, recorded in the general population. The increased occurrence of dyspnoea at incremental BMI among individuals with type 2 diabetes may reflect both cardiovascular and respiratory impairment in this high-risk patient population.

## Background

Both diabetes and respiratory disorders are highly prevalent in elderly subjects [1, 2] and often coexist in the same subjects. Almost one-half of patients with chronic bronchitis or chronic obstructive pulmonary disease (COPD) have coexisting metabolic syndrome [3, 4] and about 20% have diabetes [5]. COPD has been suggested to be a risk factor for diabetes [6, 7], but also the reverse has been reported [8]. As these two conditions both present low-grade chronic inflammation [9], they have been recently enclosed in a common general entity, also referred to as “chronic systemic inflammatory syndrome” [10]. In addition, disturbances of glucose metabolism have been found in asthmatic patients [11].

Metabolic syndrome and diabetes are associated with a modest, albeit statistically significant, impairment of pulmonary function in a restrictive pattern [2, 12–14]; however, it is currently still under debate whether and to what extent the prevalence of respiratory symptoms differs between individuals with and without diabetes. Therefore, the present study aimed at investigating the prevalence of respiratory symptoms in subjects with type 2 diabetes, as compared to the general population.

## Methods

### Studied samples

In the frame of the GEIRD (Gene Environment Interactions in Respiratory Diseases) study [15] two samples aged respectively 45-64 and 65-84 years, each comprising 1000 subjects and with a male to female ratio of one, were randomly selected from the general population of Verona, using local health authority registry. Between 2007 and ﻿2010 both samples were administered the GEIRD screening questionnaire, achieving a response percentage of 70.1% (676/965) and 60.7% (591/973) respectively (after excluding subjects who had died or moved). Between July 2009 and September 2010 the same questionnaire was given to a convenience sample of outpatients with type 2 diabetes, consequently recruited in the local Diabetes Centre (response percentage = 95.7%). Ninety-four and 165 outpatients with diabetes, aged respectively 45-64 and 65-84 years, were compared with 676 and 591 subjects in the same age range from the general population.

### Assessment of respiratory disorders

The screening questionnaire used to assess respiratory health was a modified version of the ECRHS (European Community Respiratory Health Survey) questionnaire [16], enquiring about socio-demographic characteristics, education level, smoking habits, outdoor exposure, respiratory symptoms, history of asthma, rhinitis, chronic bronchitis and eczema, and life impairment (GEIRD 2010; available at www.geird.org) [15].

Subjects were classified as having “current asthma” if they reported asthma attacks during the last 12 months or currently taking medicines for asthma, “ever asthma” if they reported the disease during the lifespan. Allergic rhinitis and eczema were defined respectively by a positive answer to the questions “*Do you have any nasal allergies including hay fever?*” or “*Have you ever had atopic dermatitis or eczema, confirmed by a doctor?*”. Chronic bronchitis was assumed if the subject answered affirmatively to the question “*Have you had cough and phlegm on most days for a minimum of three months a year and for at least two successive years?*”.

Self-reported doctor-diagnosed asthma or COPD were also considered, defined respectively by a positive answer to the questions “*Was asthma diagnosed by a doctor?*” or “*Have you ever been told by a doctor that you have or had chronic bronchitis, chronic obstructive pulmonary disease (COPD) or emphysema*? ”.

Modified Medical Research Council (mMRC) grade2 dyspnoea was assumed when the subject reported to “*walk slower than contemporaries on level ground because of breathlessness*”, or to “*stop for breath when walking at own pace”.*

### Assessment of exposures

With regard to the smoking habits, subjects were classified as follows: 1) current smokers, if they reported to have smoked at least one cigarette per day or one cigar per week for 1 year, and also during the last month; 2) ex-smokers, if they had smoked at least one cigarette per day or one cigar per week for 1 year, but not in the last month; 3) never smokers, otherwise. A previous study conducted in Verona identified a good agreement (Cohen’s k = 0.93) between self-reported smoking behavior and serum cotinine levels in participants to the ECRHS I [17].

Heavy vehicle traffic exposure was assessed by the question “*How often do heavy vehicles (e.g. trucks/buses) pass your house ?*”, whose possible answers were “*never/ seldom/ frequently/ constantly*”. In Italian participants to the ECRHS II, a good relation was found between self-reported level of heavy vehicle traffic exposure and NO2 outdoor concentration, measured by passive sampling tubes outside participants’ homes [18].

### Assessment of body-mass index and glycated haemoglobin

Information on body-mass index (BMI) and glycated haemoglobin (HbA1c) was available, respectively, in 146 (56.4%) and 179 (69.1%) individuals with type 2 diabetes. BMI was calculated by objectively measuring height (with a Harpenden stadiometer) and weight (with a properly calibrated scale), and by dividing weight in kilograms by the square of height in metres. A venous blood sample was drawn in the morning after an overnight fast. Haemoglobin A1c was measured by a high-performance liquid chromatography analyzer (Bio-Rad Diamat, Milan, Italy); the upper limit of normal for the laboratory was 5.8%.

Patients were classified according to BMI categories as normoweight (BMI <25 Kg/m^2^), overweight (25-29 Kg/m^2^) or obese (BMI ≥30 Kg/m^2^). Un-satisfactory glycaemic control was considered at HbA1c ≥7% and patients were classified accordingly in two categories (satisfactory vs. sub-optimal glycaemic control).

### Statistical analyses

Significance of differences between individuals with type 2 diabetes and the general population samples of the same age was evaluated by Fisher’s exact test for categorical variables, by chi-square test for trend for ordinal variables and by Wilcoxon-Mann-Whitney non-parametric test for continuous variables.

Significant differences in respiratory symptoms, detected in univariable analysis, were verified in multivariable analysis. The latter was accomplished by logistic regression models, where respiratory symptoms, taken one at a time, were the response variable, diabetes the explanatory variable, and sex, age (45-54 (reference), 55-64, 65-74, 75-84 years), education level (primary school (reference)/ secondary school/ high school or University), smoking habits (never (reference)/ past/ current smoker) and heavy vehicle traffic exposure (constantly/frequently exposed vs. seldom/never exposed) the potential confounders. Standard errors of ORs were adjusted for intra-sample correlation.

In people with type 2 diabetes, statistical significance of the influence of BMI (normoweight/ overweight/ obese) or HbA1c (satisfactory/ sub-optimal) on respiratory symptoms was evaluated by Fisher’s exact test, and when the association was significant in univariable analysis, a further evaluation was accomplished by a logistic regression model, controlling for sex, age (65-84 vs. 45-64 years), smoking habits (past/current vs. never smoker).

## Results

The demographic characteristics, education level, smoking habits and traffic exposure of the samples under studies are shown in Table 1. People with type 2 diabetes had a lower proportion of women than the general population in the age range 45-64 years. Individuals with diabetes of all ages had attained a lower education level and were more exposed to heavy vehicle traffic than their coevals from the general population. Smoking habits did not significantly differ between people with type 2 diabetes and the general population, although the former presented a slightly higher proportion of ex-smokers.

**Table 1:** Demographic characteristics, education level and exposure to smoking or heavy vehicle traffic in the study samples

	45-64 years			65-84 years		
	General population	Diabetic patients	*p* value	General population	Diabetic patients	*p* value
	(*n* = 676)	(*n* = 94)		(*n* = 591)	(*n* = 165)	
Female (%)	**360 (53.3)**	**37 (39.4)**	**0.015**	261 (44.2)	66 (40.2)	0.375
Age (mean ± SD)	55.3 (**±** 5.8)	56.1 (**±** 5.8)	z = −1.30, *p* = 0.193	73.3 (**±** 5.5)	73.2 (**±** 5.5)	z = 0.20, *p* = 0.842
Education level (%)			**z = −4.73,** ***p*** **< 0.001**			**z = −6.58,** ***p*** **< 0.001**
Primary school	**86 (15.6)**	**29 (31.5)**		**258 (44.2)**	**119 (74.8)**	
Secondary school	**186 (33.6)**	**36 (39.1)**		**162 (27.7)**	**24 (15.1)**	
High school	**192 (34.7)**	**24 (26.1)**		**120 (20.5)**	**15 (9.4)**	
University	**89 (16.1)**	**3 (3.3)**		**44 (7.5)**	**1 (0.6)**	
Smoking habits (%)			0.418			0.560
Never-smokers	278 (42.1)	31 (34.8)		330 (57.8)	85 (55.6)	
Ex-smokers	228 (34.6)	35 (39.3)		185 (32.4)	56 (36.6)	
Current smokers	154 (23.3)	23 (25.8)		56 (9.8)	12 (7.8)	
Heavy vehicle traffic exposure			**z = −2.51,** ***p*** **= 0.012**			**z = −6.43,** ***p*** **< 0.001**
Never	**121 (22.1)**	**18 (20.2)**		**144 (25.1)**	**15 (9.3)**	
Seldom	**236 (43.1)**	**32 (36.0)**		**227 (39.6)**	**59 (36.4)**	
Frequently	**113 (20.7)**	**11 (12.4)**		**128 (22.3)**	**30 (18.5)**	
Constantly	**77 (14.1)**	**28 (31.5)**		**74 (12.9)**	**58 (35.8)**	

*P* values were computed by Fisher’s exact test for sex and smoking habits, chi-square test for trend for education and exposure to heavy vehicle traffic and Wilcoxon-Mann-Whitney non-parametric test for age. Significant results are highlighted in bold

Individuals with diabetes of all ages more frequently reported dyspnoea limiting walking pace (modified MRC grade 2 dyspnoea) than their coevals (*p* < 0.001) (Table 2). Compared to the general population, self-reported chronic cough/phlegm was more common among individuals with diabetes aged 45-64 years (*p* = 0.017), while self-reported eczema tended to be more common, although not significantly, among those aged 65-84 years (*p* = 0.087).

**Table 2:** Prevalence of respiratory disorders in the study samples

	45-64 years			65-84 years		
	General population	Diabetic patients	*p* value	General population	Diabetic patients	*p* value
	(*n* = 676)	(*n* = 94)		(*n* = 591)	(*n* = 165)	
Ever asthma (%)	58 (8.8)	9 (9.7)	0.702	42 (7.5)	10 (6.2)	0.730
Current asthma (%)	39 (5.8)	6 (6.5)	0.814	25 (4.4)	8 (4.9)	0.830
Diagnosed asthma (%)	43 (6.6)	6 (6.7)	1.00	27 (4.9)	5 (3.1)	0.513
Allergic rhinitis (%)	125 (18.7)	15 (16.5)	0.668	75 (13)	24 (15.1)	0.513
Eczema (%)	89 (13.6)	14 (15.6)	0.626	70 (12.8)	28 (18.3)	0.087
Chronic cough/phlegm (%)	**108 (16.7)**	**24 (27.6)**	**0.017**	134 (24.6)	41 (27.9)	0.454
Diagnosed COPD (%)	41 (6.2)	7 (7.9)	0.496	78 (14.0)	18 (11.8)	0.593
mMCR grade2 dyspnoea (%)	**52 (7.9)**	**20 (23)**	**<0.001**	**77 (13.7)**	**47 (30.9)**	**<0.001**

*P* values were computed by Fisher’s exact test. Significant results are highlighted in bold

**Fig. 1:**
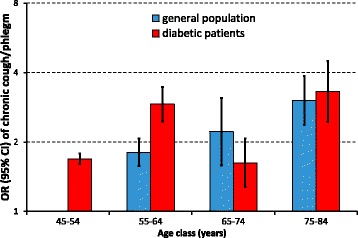
Odds ratios (ORs) of reporting chronic cough/phlegm in the general population (*blue columns*) and in individuals with type 2 diabetes (*red columns*) as a function of age class. Columns are ORs, bars are 95% confidence intervals. ORs were computed by a logistic regression model, controlling for sex, age, education level, smoking habits and exposure to heavy vehicle traffic

**Fig. 2:**
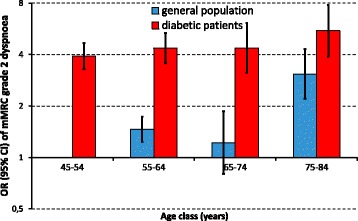
Odds ratios (ORs) of reporting mMRC grade 2 dyspnoea in the general population (*blue columns*) and in individuals with type 2 diabetes (*red columns*) as a function of age class. Columns are ORs, bars are 95% confidence intervals. ORs were computed by a logistic regression model, controlling for sex, age, education level, smoking habits and exposure to heavy vehicle traffic

Significant differences recorded in univariate analysis were verified in multivariable analysis, where a remarkable difference was detected in age-related pattern of respiratory disorders between the general population and people with type 2 diabetes. In the general population, the risk of reporting chronic cough/phlegm (Fig. 1) or mMRC grade 2 dyspnoea (Fig. 2) increased three-fold from 45- 54 years to 75-84 years. Among people with type 2 diabetes the risk of these respiratory disorders was particularly high in the younger age classes, but the increasing trend across incremental age categories was less pronounced. As a consequence, individuals with diabetes presented a greater risk of respiratory disorders than the general population in the younger age classes, while the difference tended to fade with ageing. With respect to the general population, the OR of mMRC grade 2 dyspnoea among individuals with diabetes was nearly four (3.92, 95% CI 3.28-4.68; *p* < 0.001) in the age class 45-54 years, and decreased to 1.79 (1.68-1.91; *p* < 0.001) in the age class 75-84 years (Fig. 2). Similarly, the ORs of chronic chough/phlegm decreased from 1.69 (1.60-1.78; *p* < 0.001) to 1.09 (1.03-1.16; *p* = 0.006) across the same age ranges (Fig. 1). As a consequence, the interaction between age class and diabetes was significant for both respiratory disorders.

With regard to the other variables included in the model, constant/frequent exposure to heavy vehicle traffic was associated with a significant increase in the risk of both chronic cough/phlegm (OR = 2.15, 1.85-2.48; *p* < 0.001) and mMRC grade 2 dyspnoea (OR = 1.67, 1.36 – 2.06; *p* < 0.001), with respect to absent/rare exposure. Moreover, chronic cough/phlegm was more frequently reported by past smokers (OR = 1.47, 1.09-1.97; *p* = 0.011) and current smokers (OR = 3.09, 2.23-4.30; *p* < 0.001), as compared to never smokers, while mMRC grade 2 dyspnoea was more frequently reported by women (OR = 2.02, 1.14–3.59; *p* = 0.016) with respect to men.

Most subjects with diabetes were overweight (48%) or obese (33%) and more than a half (61%) did not achieve a satisfactory glycemic control, having HbA1c ≥7%. Median BMI was 28.0 Kg/m^2^ (interquartile range 25.5-32.0) and median HbA1c was 7.25% (6.4-8.3). Of note, the proportion of self-reported dyspnoea among people with diabetes increased across incremental BMI categories from 15.4 to 25.4% and further to 41.3%, respectively, in normoweight, overweight and obese outpatients (*p* = 0.048). When controlling for sex, age, smoking habits by a logistic regression model, the resulting OR of grade 2 dyspnoea was 2.07 (0.58-7.38; *p* = 0.262) in overweight patients with type 2 diabetes and 4.51 (1.22-16.70; *p* = 0.024) in obese patients, with respect to normoweight patients. Glycated haemoglobin was significantly associated with eczema: patients with HbA1c <7% more frequently reported eczema than the other patients (28% vs. 12%; *p* = 0.013), and in the multivariable logistic model they presented an OR of self-reported eczema of 3.2 (1.3-8.1; *p* = 0.013) with respect to patients with HbA1c ≥7%. No other significant association between BMI or HbA1c and respiratory disorders in individuals with type 2 diabetes was detected.

## Discussion

The main results of the present study are:

First, individuals with type 2 diabetes more frequently reported chronic cough and phlegm, and grade 2 dyspnoea as compared to the general population of the same age, although presenting similar smoking habits. These differences tended to decrease at older ages, thus suggesting that diabetes induces an accelerating ageing of the lung.

Second, grade 2 dyspnoea was most often reported by obese outpatients with type 2 diabetes.

Third, the prevalence of asthma or allergic rhinitis was not significantly associated with diabetes. Eczema tended to be more prevalent in individuals with type 2 diabetes within the age range 65-84 years, although the association was not statistically significant.

The most remarkable finding of the present study was a three-to-four fold increase in dyspnoea observed in individuals with type 2 diabetes with respect to the general population. Accordingly, available literature supports the notion that the lung is a target organ for diabetic injury [19] and that type 2 diabetes is associated with reduced lung volumes and airflow limitation [20]. Usually, a pattern of modest lung restriction and reduced diffusion capacity has been described in individuals with type 2 diabetes [13, 14, 21, 22]. However, some studies have also linked obstructive diseases, in particular COPD, to metabolic syndrome or impaired glucose tolerance [10, 23].

In the present study, the prevalence of diagnosed COPD did not differ between individuals with type 2 diabetes and coevals from the general population. However, chronic cough/phlegm, which was once considered the first stage of the disease (GOLD stage 0) [24], was more commonly reported by people with diabetes.

Although dyspnoea is the distinctive hallmark of most respiratory diseases, obesity or impairment of the cardiovascular system may be responsible for the increased prevalence of dyspnoea among individuals with type 2 diabetes. However, impairment of both cardiovascular and respiratory systems may frequently coexist in the same subject. The recent ECLIPSE epidemiological study found that diabetes was associated with higher MRC dyspnoea scores and reduced 6-min walk distance and that comorbidities of heart disease and diabetes correlated with increased systemic inflammation [25].

Individuals with type 2 diabetes self-reported a higher level of exposure to heavy vehicle traffic than the general population, and this difference could partly explain the higher prevalence of dyspnoea and chronic cough/phlegm among people with type 2 diabetes. However, the association between type 2 diabetes and respiratory disorders persisted even after controlling for traffic exposure in a multivariable model.

In the present study an increased prevalence of eczema was observed among older individuals with type 2 diabetes. However, although an association between diabetes and eczema was expected on the basis of current literature, this figure did not reach significance, possibly due to limited statistical power. Indeed, topical steroids are often used for long-term control of eczema despite contraindications, and this could not only worsen eczema, but also elicit insulin resistance. An altered glucose metabolism occurs in nearly half of glucocorticoid-treated patients, even after treatment reduction or withdrawal [26]. Moreover, a larger amount of steroid could be absorbed during topic treatment in elderly individuals, due to reduced skin thickness [27]. Alternately, insulin resistance status could induce eczema, by decreasing the expression of Δ-6-desaturase [28], an hepatic enzyme involved in the synthesis of gamma-linolenic acid, whose levels are reduced in atopic eczema [29].

Asthma-like symptoms have been reported to be more frequent in adults with metabolic syndrome [30] or with insulin resistance, a basic alteration of type 2 diabetes [31]. Other authors have argued that insulin itself may increase the contractility of airway smooth muscle cells, thus contributing to bronchial hyper-responsiveness [21]. However, these findings were not supported by the present research, as the prevalence of diagnosed or self-reported asthma was similar in both individuals with type 2 diabetes and in the general population.

### Pathophysiologic mechanisms

Hyperglycemia could trigger inflammatory response, thus leading to structural modification of pulmonary tissue and impaired lung function [9]. Moreover, lung function volumes have been shown to be inversely associated with insulin resistance [31].

On the other hand, it could be also hypothesized that diabetes can worsen or even accelerate the derangement of respiratory function by inducing structural alterations [9, 32]. In fact, the pulmonary alveolar-capillary network is a large microvascular unit that may also be affected by diabetic microangiopathy [33]. Human autopsy studies in subjects with diabetes have observed cell proliferation with hypertrophy of interstitial matrix, increased thickness of the bronchial, alveolar and capillary basement membranes and an accompanying collapse of the alveolar space [34]. In this regard, a recent study reported a significant reduction of carbon monoxide diffusing capacity in individuals with diabetes, as compared to their counterparts without diabetes [35]. This structural alteration may also be related to the increased risk of individuals with diabetes to be hospitalized for pneumonia [35, 36].

### Strengths and limitations

A strength of this study was the use of a standardized internationally-validated questionnaire to assess respiratory health. In addition, when assessing the impact of diabetes on respiratory symptoms, samples from the general population of the same age were considered as reference.

Smoking is the main risk factor for respiratory diseases in the Western world, being responsible for up to 80-90% of COPD cases [37], but it also enhances the risk of developing type 2 diabetes [38–40]. Hence, smoking status may act as a confounder of the relation between diabetes and respiratory health. However, in the present study smoking habits were not significantly different between individuals with type 2 diabetes and their counterparts from the general population. Moreover, multivariable analysis was adjusted for self-reported smoking history, as well as for heavy vehicle traffic exposure.

However, some limitations should also be acknowledged. First, information on respiratory health was collected by a self-administered questionnaire without direct assessment of respiratory function. Second, response percentage was higher among individuals with type 2 diabetes (95.7%) than in the general population samples (70.1 and 60.7%, respectively, in people aged 45-64 and 65-84 years). As people with respiratory disorders tend to be early responders to self-administered questionnaires [41], a reduction in response percentage may have led to overestimate the prevalence of respiratory symptoms in the general population, thus partly masking the difference between individuals with type 2 diabetes and the general population. Third, the prevalence of type 2 diabetes can be expected to be around 10% in the general Italian population aged 45-84 years [1, 42]. However, individuals with diabetes could not be excluded from general population samples, as the GEIRD screening questionnaire did not enquire about this disease. This probably could have also led to underestimate differences in respiratory disorders between subjects with diabetes and the general population. Finally, the cross-sectional design of the present study does allow to detect associations, such as between respiratory disorders and type 2 diabetes, but not to infer the causal relation involved.

## Conclusions

In conclusion, our data confirmed the evidence of an increased prevalence of respiratory symptoms (dyspnoea, chronic cough/phlegm) in individuals with type 2 diabetes, when compared to the general population of the same age. This difference was larger in the age range 45-64 years than at older ages, thus suggesting that diabetes may anticipate the lung ageing process. Therefore, our results may be relevant to the practicing physicians as they highlight the potential clinical utility of an early screening for respiratory diseases in individuals with type 2 diabetes. When deemed necessary, the prompt initiation of specific treatments for respiratory diseases would be advisable by comprehensively accounting for the accompanying multi-morbidities and modifiable risk factors.

## Abbreviations

BMI: Body Mass Index
COPD: Chronic Obstructive Pulmonary Disease
ECRHS: European Community Respiratory Health Survey
GEIRD: Gene Environment Interactions in Respiratory Diseases
HbA1c: Glycated Haemoglobin
mMRC: Modified Medical Research Council grade 2 dyspnoea
